# Analysis of SOD2 rs4880 Genetic Variant in Patients with Alzheimer’s Disease

**DOI:** 10.3390/cimb44100302

**Published:** 2022-09-21

**Authors:** Vasileios Siokas, Polyxeni Stamati, Georgia Pateraki, Ioannis Liampas, Athina-Maria Aloizou, Daniil Tsirelis, Anastasia Nousia, Markos Sgantzos, Grigorios Nasios, Dimitrios P. Bogdanos, Efthimios Dardiotis

**Affiliations:** 1 Laboratory of Neurogenetics, Department of Neurology, University Hospital of Larissa, Faculty of Medicine, School of Health Sciences, University of Thessaly, 41100 Larissa, Greece; 2 Department of Speech and Language Therapy, University of Ioannina, 45500 Ioannina, Greece; 3 Department of Rheumatology and Clinical Immunology, University Hospital of Larissa, Faculty of Medicine, School of Health Sciences, University of Thessaly, 41100 Larissa, Greece

**Keywords:** AD, Alzheimer’s disease, SOD2, oxidative stress, variant, rs4880, genetics

## Abstract

A few gene loci that contribute to Alzheimer’s Disease (AD) onset have been identified. Few studies have been published about the relationship between SOD2 rs4880 single nucleotide variant and AD, revealing inconsistent results. Therefore, the aim of the current study is to further examine the role of the SOD2 rs4880 in AD. We performed a case-control study with a total of 641 subjects (320 patients with probable AD, and 321 healthy controls). The statistical analysis was performed assuming five genetic models. The threshold for statistical significance was set at 0.05. The results revealed no association between SOD2 rs4880 and AD in any of the assumed genetic models that were examined [log-additive OR = 0.95 (0.76–1.19), over-dominant OR = 1.15 (0.85–1.57), recessive OR = 0.85 (0.59–1.22), dominant OR = 1.03 (0.72–1.47), and co-dominant OR1 = 1.10 (0.75–1.60) and OR2 = 0.90 (0.58–1.40)]. Adjustment for sex and subgroup analyses based on sex did not reveal any statistically significant results either. Based on our findings, SOD2 rs4880 does not appear to play a determining role in the risk of developing AD. Larger studies are warranted to elucidate the connection between rs4880 and AD.

## 1. Introduction

Alzheimer’s disease (AD) is the most common form of dementia, as it constitutes 60–70% of dementia cases [1,2]. Human aging is a strong predisposing factor for AD with several cognitive, anatomical, and neurophysiological changes occurring with increasing age [3]. In fact, aging is strongly associated with AD, in a way that numerous people will manifest characteristics of the disease, and so the increasing age will inevitably increase the prevalence of AD [4]. AD is considered a neurodegenerative disease with chronic progression, starting with impairments in the ability to encode and save new memories [5].

Regarding the epidemiology of AD higher rates are reported in southern European countries compared to northern European countries, while women show a higher prevalence compared to men [6]. Inevitably, the data about the prevalence of people in preclinical stages of AD are scarcer, although 15–20% of people older than 64 years have Mild Cognitive Impairment (MCI), 14.9% of which are going to develop dementia in a couple of years [7].

A few genetic loci have already been identified that contribute to AD onset. However, it is necessary to identify new genetic risk factors via genome-wide association and candidate gene association studies, which have already revealed the complex nature of the genetics involved in AD [8]. Early-onset AD is categorized in most cases as early-onset familial Alzheimer’s disease (EOFAD), developed at a young age (<65 years), and represents 1–5% of total AD cases [9,10]. Other than the familial form, the sporadic form is responsible for 95% of AD cases [11,12], with the ε4 allele of the APOE gene exhibiting the strongest genetic association with sporadic AD [11,13]. Epigenetic mechanisms and environmental factors may also play a potential role in AD [11,14,15].

AD’s pathophysiological mechanisms have not been fully elucidated, though they mostly encompass amyloid-beta peptide accumulation in brain tissues and changes in the phosphorylation of Tau proteins in neurons which cause cytoskeletal impairments [16]. Moreover, many reviews refer to oxidative stress as an important contributor to the development of different neurodegenerative diseases, AD included [17]. The human brain is extremely sensitive to oxidative stress due to its elevated rate of oxygen metabolic activity, high levels of polyunsaturated fatty acids, a very oxidizable substrate, and low amounts of antioxidant systems compared to other organs [18,19]. Oxidative stress aggravates the production and accumulation of toxic amyloid plaques, as well as the phosphorylation of tau protein, the hallmarks of the pathogenesis of AD [20]. In addition, mitochondrial stress, and stress of the endoplasmatic reticulum (ER) have also been related to the pathophysiology of AD.

Superoxide dismutase 2 (SOD2) is a manganese-containing enzyme belonging to the major antioxidant defense system of the organism. It is synthesized in the cytoplasm and is directly transported to the mitochondria where it protects the cell by catalyzing O_2_- generated by the respiratory chain, into H_2_O_2_ and molecular oxygen [21]. It possesses an essential role in maintaining mitochondrial proper function ensuring cellular survival [21,22]. 

The SOD2 gene is located in chromosome 6 in the region q25 [23,24]. In the available bibliography, studies have so far explored the relationship between the SOD2 rs4880 genetic variant and AD, and have yielded inconsistent results [25,26,27]. Therefore, the aim of the current study is to further examine the role of the SOD2 rs4880 in AD.

## 2. Materials and Methods

### 2.1. Study Design, Ethics and Informed Consent

We conducted a case-control study in order to examine the effect of the SOD2 rs4880 genetic variant on the risk of AD. Written informed consent was granted from all individuals (or their close relatives when necessary) that were included in the present study. The Ethics Committee of the University General Hospital of Larissa approved the research protocol (132/17-06-2015). 

### 2.2. Study Population

The current case-control study includes the same sample from previously published articles [28,29,30,31]. In brief, we prospectively recruited patients with AD from the outpatient and inpatient clinics of the Neurology Department of the General University Hospital of Larissa, a tertiary referral institution located in Central Greece. The National Institute of Neurological and Communicative Disorders and Stroke/Alzheimer Disease and Related Disorders Association (NINCDS/ADRDA) criteria [32] were applied by a Senior Neurologist, in order for the diagnosis of probable AD to be set. The control group consisted of healthy volunteers with normal mini-mental state examination (MMSE) scores that did not fulfill the criteria for MCI and without any reported medical history record.

### 2.3. Molecular Genetics and Laboratory Techniques

Leucocytes derived from peripheral blood samples from each participant were used, for nuclear DNA extraction. For this procedure, we applied the salting out method. After the isolation of the genetic material, we proceeded to genotype all the samples for the SOD2 rs4880 genetic variant. For the genotyping implementation, we applied the TaqMan allele-specific discrimination assay (Thermo Fisher Scientific, Waltham, MA, USA) on an ABI PRISM 7900 Sequence Detection System, while the SDS software (SDS 2.4) (Applied Biosystems, Foster City, CA, USA) was used to analyze the results. The entire method (PCR steps, enzyme activation, denaturation, and annealing/extension) has been previously extensively described [33]. Τo minimize any potential bias, the genotyping was carried out exclusively by personnel who were blinded to the clinical status of the samples.

### 2.4. Quality Assessment Procedures

Aiming to enhance the quality of the genotyping results and provide robustness, we re-genotyped a randomly selected 10% of the samples, leading to a 100% concordance with the initial genotyping results. Additionally, we set the threshold of the genotypic call rate (percentage of successfully genotyped samples) at 95%. Finally, we examined the genotyping results for any deviation from the Hardy–Weinberg Equilibrium (HWE), via Chi-squared test calculation [34]. 

### 2.5. Statistical Analysis and Outcome Measures

The outcome of the current study was to explore any potential association between the SOD2 rs4880 and AD. The demographic characteristics are described as means ± standard deviation (SD) for continuous variables, and as n and/or percentages for categorical variables. The statistical power of the sample size was calculated using the CaTS Power Calculator for Genetic Studies (Center for Statistical Genetics, University of Michigan, Ann Arbor, MI, USA). The total number (n) and the percentages for allelic and genotypic distribution, in AD cases, in healthy controls, and the whole sample have been calculated. The effect size of the association between the SOD2 rs4880 and the AD was expressed in the terms of the odds ratios (ORs) and their precision [95% confidence intervals (CIs)]. The implementation of this analysis was made with the application of the SNPStats software (https://www.snpstats.net/, accessed on 10 April 2022) [34], assuming the following five genetic models (co-dominant, dominant, recessive, over-dominant, and log-additive). Subgroup analysis based on sex was also performed. The ‘T’ was considered as the reference allele and the ‘C’ as the alternative one for all of the analyses, with the exception of the female subgroup analysis where the “C” was considered as the reference. The *p*-value < 0.05 was set as the statistically significant threshold.

## 3. Results

We genotyped an initial cohort of 654 subjects (327 patients with AD and an equal number of healthy controls). The overall genotype call rate was 98.01% (641/654). After excluding the subjects that failed to be genotyped, our cohort consisted of 320 patients with AD (33.8% male, mean age of blood collection ± SD = 78.88 ± 8.63 years) and 321 healthy controls (55.8% male, mean age ± SD = 69.72 ± 3.03). The basic demographic and clinical characteristics of the AD and healthy control groups are presented in Table 1.

Moreover, we did not detect any deviation from the HWE in either AD patients or the healthy controls (*p* = 0.37 and *p* = 0.82, respectively). Our sample had a power of 80.3 to detect a significant association (*p* < 0.05) between the SOD2 rs4880 variant and AD, with the frequency of the C allele equal to 50%, a prevalence of AD equal to 37/100,000, and a relative risk of 1.37 for the multiplicative mode of inheritance.

The allelic distribution for the minor allele (G) was 49% in the group of patients with AD, and 50% in the healthy controls group. Moreover, the genotypic distribution was 79 (25%), 169 (53%), and 72 (22%) for the T/T, T/C, and C/C in the group of patients with AD. The values in the healthy control group were 81 (25%), 158 (49%), and 82 (26%), respectively. The SOD2 rs4888 variant total numbers of the alleles and genotypes for all subjects, the healthy controls, and for the patients with AD are presented in Table 2.

No association (*p* > 0.05) was found between the SOD2 rs4880 and AD in any of the examined genetic models of inheritance [log-additive OR = 0.95 (0.76–1.19), over-dominant OR = 1.15 (0.85–1.57), recessive OR = 0.85 (0.59–1.22), dominant OR = 1.03 (0.72–1.47), and co-dominant OR1 = 1.10 (0.75–1.60) and OR2 = 0.90 (0.58–1.40)]. Adjustment for sex could not reveal any statistically significant results (*p* > 0.05) [log-additive OR = 0.91 (0.73–1.15), over-dominant OR = 1.15 (0.83–1.58), recessive OR = 0.81 (0.56–1.16), dominant OR = 0.98 (0.68–1.42), and co-dominant OR1 = 1.05 (0.71–1.55) and OR2 = 0.84 (0.53–1.22)]. The respective results are depicted in Table 3.

Subgroups analysis based on sex revealed no association (*p* > 0.05) between rs4880 and AD. The SOD2 rs4888 variant total numbers and frequencies of the alleles and genotypes based on sex are depicted in Table S1 (see Supplementary material) for male participants and in Table S2 for female participants. Results regarding the association analysis between rs4880 and AD are presented in Table S3 for the male participants and in Table S4 for the female participants.

## 4. Discussion

In the present case-control study, we recruited a large number of patients with AD as well as healthy controls and genotyped them for the SOD2 rs4880 variant. We did not detect any association between this variant and the risk of developing AD. To the best of our knowledge, this is the first study to investigate this variant and its possible correlation with AD in a sample of Greek origin.

The SOD2 gene is located in chromosome 6 in the region q25 [23,24]. Almost 190 genetic variations variants have been described in the SOD2 gene until now, and have been involved in breast cancer, diabetes mellitus, dyslipidemia, and other diseases [24]. The SOD2 gene encodes the SOD2, a manganese-containing enzyme that belongs to the major antioxidant defense system. Impaired SOD2 enzymatic activity leads to increased oxidative stress [35]. The rs4880 (C47T) is a missense variant in exon 2 located at Chromosome 6:159692840 (http://www.ensembl.org/Homo_sapiens/Variation/Explore?r=6:159692340159693340;v=rs4880;vdb=variation;vf=167107369, accessed on 10 April 2022). When the T allele is present, the amino acid valine (Val) is encoded at codon 16, while the amino acid alanine (Al) is encoded instead in the case of the C allele [36,37]. The T allele of the SNP rs4880 results in structural alterations in the mitochondrial targeting domain of SOD2, leading to its less efficient post-transcriptional transport into the mitochondrion and decreased potential in neutralizing superoxide anions [37].

Studies have so far explored the relationship between SOD2 rs4880 and AD have yielded inconsistent results [25,26,27]. The study of Wiener et al. (2007) revealed significant indication of an association between rs4880 and AD, using family-based association tests. [25]. The case-control study of Spisak et al. (2014) did not prove any association between rs4880 and AD [26]. Finally, the study of Gamarra et al. (2015) showed that the SOD2 rs4880 in combination with APOEε4 allele carriage increases the risk for MCI, while it also increases the risk for AD compared to MCI [27].

Oxidative stress represents an imbalance between oxidants and antioxidants in favor of the oxidants, leading to severe damage to proteins, lipids, DNA, and RNA of the cells at several levels [38]. The main representatives of oxidative stress are the reactive oxygen species (ROS) and reactive nitrogen species (RNS), products of normal cellular metabolism, which can play both beneficial and catastrophic roles to the cell and the organism [39,40]. In low amounts, they act as a part of the immune system against infectious agents, contribute to the cellular signaling system, and activate several important metabolic pathways of the cellular metabolism [39,40]. On the other hand, when they are found in excessively high amounts, as in oxidative stress, they cause severe biologic damage inducing cellular death [41].

Mitochondria, as a major source of ROS production, are significantly vulnerable to oxidative stress and their malfunction contributes to the course of aging and neurodegenerative processes [20,42,43]. Many studies have shown a number of changes in the mitochondria in the brains of patients with AD, such as smaller size, lower number, impaired mitochondrial transport to regions of high energy demands, and altered mitochondrial fission–fusion protein dynamics, all leading to loss of synaptic plasticity and normal neuronal function, contributing to neurodegeneration [44,45,46].

AD represents a major example of the protein folding disease pattern. The ER is the center of folding and transport of newly formed proteins in the cell membrane, while it also keeps a key role in the maintenance of calcium homeostasis [47]. Any disturbance in the normal protein folding process may lead to the accumulation of misfolded or unfolded proteins in the ER. In response to that, an important pathway called the unfolded protein response (UPR) is activated in order to protect the cell [48]. Many studies have confirmed that UPR is involved in the early stages of AD, and ER stress markers have been described in tissues derived from patients with AD. Additionally, a self-implying relationship has been shown between hyperphosphorylation of tau protein and UPR activation, leading to a vicious cycle of neuronal degeneration [49,50]. Similarly, toxic aβ oligomers, through a drastic disturbance of ER calcium homeostasis, produce even more altered protein folding and oxidative stress, leading to cell death [51].

For this important role, studies have shown that SOD2 deficiency causes mitochondrial damage in cells with high levels of oxidative metabolism, like neurons, hemopoietic, and hepatic cells [52]. Impaired SOD2 activity has been related to several diseases such as cancer, particularly ovarian and breast cancer, diabetes mellitus, and neurodegeneration, including AD [52,53]. It seems that SOD2 is an important factor in the pathogenesis of the disease since it has a key role in repairing oxidative damage. Additionally, SOD2 may regulate neuroinflammation by controlling the activation of microglia. Interestingly, studies indicate that at the beginning of the inflammatory response activated microglia regulate the activation of SOD2 and at the end of inflammation increased SOD2 inactivates microglia, protecting the cell from the oxidative stress of chronic inflammatory processes [54].S

Moving on, we must acknowledge the limitations of the present study. Firstly, some of the patients’ data have been based on patients’/caregivers’ self-reporting, as most of them were hospitalized with advanced AD stage. Therefore, it was not possible to include the precise age at AD onset in the analyses. Consequently, we performed adjusted analyses only for sex without including additional potential predisposing or precipitating AD risk factors (genetic and not-genetic, especially the APO ε4 carriage status) in regression statistical models. Therefore, the possibility that our results are significantly affected by the latent effect of uncontrolled co-founders cannot be totally excluded. This could partially explain the lack of an association in our study, as the SOD2 rs4880 variant may be an additional risk factor for the development of an amnestic syndrome. This was reciprocated in the study of Gamarra et al. (2015), where SOD2 rs4880 T allele carriage was shown to increase the risk of amnestic MCI patients carrying APOEε4 [27]. Following the previous limitation, we included subjects without screening for major AD-linked genes [8]. Finally, additional analyses correlating SOD2 rs4880 genotyping data with other phenotypes (e.g., age at AD onset, disease duration, disease progression, and MMSE score) would have provided more robustness to our conclusions.

## 5. Conclusions

In conclusion, we provide these first data from a Greek population regarding the SOD2 rs4880 variant and AD. It is crucial that additional studies be performed in order to elucidate the role of SOD2 rs4880 in AD. More precisely, large multicenter studies, possibly also measuring the enzymatic activity of SOD2 and also adjusting for co-founders that confer susceptibility to AD in order for the attributable risk of this variant to AD to be fully elucidated are needed.

## Figures and Tables

**Table 1:** Demographic characteristics of healthy controls and patients with AD.

	Healthy Controls (n = 321)	AD (n = 320)
Age, years mean (SD)	69.72 (3.03)	78.88 (8.63) *
Sex n (%)		
Male	179 (55.8)	108 (33.8)
Female	142 (44.2)	212 (66.3)

AD, Alzheimer’s Disease; SD, standard deviation. * age at blood collection.

**Table 2:** Allelic and genotype total numbers for SOD2 rs4880 in healthy controls, in AD cases, and whole sample.

Variant	Genotypes/ Alleles	Healthy Controls (n = 321)	AD (n = 320)	Whole Sample (n = 641)
rs4880		n	n	n (%)
Genotype	T/T	81	79	160
	C/T	158	169	327
	C/C	82	72	154
Allele	T	320	327	647
	C	322	313	635

SOD2 Superoxide dismutase 2; AD Alzheimer’s Disease.

**Table 3:** Single locus analysis for the association between SOD2 rs4880 and AD, in co-dominant, dominant, recessive, over-dominant, and log-additive modes.

	Unadjusted Analysis			Adjusted Analysis	
Mode	Genotype	OR (95%CI)	*p*-Value	OR (95%CI)	*p*-Value
Codominant	T/T	1.00	0.59	1.00	0.53
	C/T	1.10 (0.75–1.60)		1.05 (0.71–1.55)	
	C/C	0.90 (0.58–1.40)		0.84 (0.53–1.33)	
Dominant	T/T	1.00	0.87	1.00	0.92
	C/T-C/C	1.03 (0.72–1.47)		0.98 (0.68–1.42)	
Recessive	T/T-C/T	1.00	0.37	1.00	0.27
	C/C	0.85 (0.59–1.22)		0.81 (0.56–1.18)	
Overdominant	T/T-C/C	1.00	0.36	1.00	0.4
	C/T	1.15 (0.85–1.57)		1.15 (0.83–1.58)	
Log-additive	-	0.95 (0.76–1.19)	0.65	0.92 (0.73–1.15)	0.47

SOD2, Superoxide dismutase 2; AD, Alzheimer’s Disease; CI, confidence interval; OR, odds ratio. Adjustment was made for sex as a categorical variable.

## Data Availability

The data presented in this study are available on reasonable request from the corresponding author.

## Supplementary Materials

The following supporting information can be downloaded at: https://www.mdpi.com/article/10.3390/cimb44100302/s1. Table S1: Allelic and genotype total numbers for SOD2 rs4880 in male participants (healthy controls, in AD cases, and whole male sample); Table S2: Allelic and genotype total numbers for SOD2 rs4880 in female participants (healthy controls, in AD cases, and whole female sample); Table S3: Single locus analysis for the association between SOD2 rs4880 and AD, in co-dominant, dominant, recessive, over-dominant and log-additive mode, in male participants; and Table S4: Single locus analysis for the association between SOD2 rs4880 and AD, in co-dominant, dominant, recessive, over-dominant and log-additive mode, in female participants.
