# Supplementary material for: Analysis of SOD2 rs4880 Genetic Variant in Patients with Alzheimer’s Disease
Source: Curr Issues Mol Biol. 2022 Sep 21;44(10):4406–14. doi: 10.3390/cimb44100302 (PMC9600469; doi:10.3390/cimb44100302)
Supplement: Supplementary file 1 [file cimb-44-00302-s001.zip › cimb-1872593-supplementary.pdf]

***Table S1**. Allelic and genotype total numbers for SOD2 rs4880 in male participants (healthy controls, in AD cases, and whole male sample)*

**Variant**	**Genotypes/ Alleles**	**Healthy Controls** (n=179)	**AD** (n=108)	**Whole sample** (n=287)
rs4880		n	n	n (%)
Genotype	T/T	47	31	78
	C/T	90	54	144
	C/C	42	23	65
Allele	T	184	116	300
	C	174	100	274

SOD2 Superoxide dismutase 2; AD Alzheimer's Disease.

***Table S2**. Allelic and genotype total numbers for SOD2 rs4880 in female participants (healthy controls, in AD cases, and whole female sample)*

**Variant**	**Genotypes/ Alleles**	**Healthy Controls** (n=142)	**AD** (n=212)	**Whole sample** (n=354)
rs4880		n	n	n (%)
Genotype	T/T	34	48	82
	C/T	68	115	183
	C/C	40	49	89
Allele	T	136	211	347
	C	148	213	361

SOD2 Superoxide dismutase 2; AD Alzheimer's Disease.

Table S3. *Single locus analysis for association between SOD2 rs4880 and AD, in co-dominant, dominant, recessive, over-dominant and log-additive mode, in male participants*

**Mode**	**Genotype**	**OR (95%CI)**	**p-value**
Codominant	T/T	1.00	0.87
	C/T	0.91 (0.52-1.60)	
	C/C	0.83 (0.42-1.64)	
Dominant	T/T	1.00	0.65
	C/T-C/C	0.88 (0.52-1.51)	
Recessive	T/T-C/T	1.00	0.67
	C/C	0.88 (0.50-1.57)	
Overdominant	T/T-C/C	1.00	0.96
	C/T	0.99 (0.61-1.59)	
Log-additive	---	0.91 (0.65-1.28)	0.59

SOD2, Superoxide dismutase 2; AD, Alzheimer's Disease; CI, confidence interval; OR, odds ratio.

Table S4. *Single locus analysis for association between SOD2 rs4880 and AD, in co-dominant, dominant, recessive, over-dominant and log-additive mode, in female participants*

**Mode**	**Genotype**	**OR (95%CI)**	**p-value**
Codominant	C/C	1.00	0.45
	C/T	1.38 (0.83-2.31)	
	T/T	1.15 (0.63-2.11)	
Dominant	C/C	1.00	0.28
	C/T-T/T	1.30 (0.80-2.12)	
Recessive	C/C-C/T	1.00	0.78
	T/T	0.93 (0.56-1.54)	
Overdominant	C/C-T/T	1.00	0.24
	C/T	1.29 (0.84-1.98)	
Log-additive	---	1.08 (0.80-1.47)	0.62

SOD2, Superoxide dismutase 2; AD, Alzheimer's Disease; CI, confidence interval; OR, odds ratio.
